# Hydrogen Production by the Thermophilic Bacterium *Thermotoga neapolitana*

**DOI:** 10.3390/ijms160612578

**Published:** 2015-06-04

**Authors:** Nirakar Pradhan, Laura Dipasquale, Giuliana d’Ippolito, Antonio Panico, Piet N. L. Lens, Giovanni Esposito, Angelo Fontana

**Affiliations:** 1 Department of Civil and Mechanical Engineering, University of Cassino and Southern Lazio, Via Di Biasio, 43, 03043 Cassino, FR, Italy; E-Mails: nirakar.pradhan@gmail.com (N.P.); giovanni.esposito@unicas.it (G.E.); 2 Istituto di Chimica Biomolecolare, Consiglio Nazionale delle Ricerche, Via Campi Flegrei 34, 80078 Pozzuoli, Napoli, Italy; E-Mails: ldipasquale@icb.cnr.it (L.D.); gdippolito@icb.cnr.it (G.I.); 3 Telematic University Pegaso, piazza Trieste e Trento, 48, 80132 Naples, Italy; E-Mail: anpanico@unina.it; 4 UNESCO-IHE Institute for Water Education, Westvest 7, 2611-AX Delft, The Netherlands; E-Mail: piet.lens@wur.nl

**Keywords:** thermophilic bacteria, fermentation, hydrogen, lactic acid, carbon dioxide, biomass, renewable energy, process kinetics, energy carrier, green-house gas

## Abstract

As the only fuel that is not chemically bound to carbon, hydrogen has gained interest as an energy carrier to face the current environmental issues of greenhouse gas emissions and to substitute the depleting non-renewable reserves. In the last years, there has been a significant increase in the number of publications about the bacterium *Thermotoga neapolitana* that is responsible for production yields of H_2_ that are among the highest achievements reported in the literature. Here we present an extensive overview of the most recent studies on this hyperthermophilic bacterium together with a critical discussion of the potential of fermentative production by this bacterium. The review article is organized into sections focused on biochemical, microbiological and technical issues, including the effect of substrate, reactor type, gas sparging, temperature, pH, hydraulic retention time and organic loading parameters on rate and yield of gas production.

## 1. Introduction

Anaerobic digestion of organic material is regarded as a potential method for hydrogen (H_2_) production from biomass [1,2]. Besides simple carbohydrates (e.g., glucose) or polymers such as starch and cellulose, the process utilizes a wide range of organic compounds as substrate, including organic wastes and agro-industrial matrices [3,4,5,6,7,8,9]. Considering that such residues are abundant, cheap, renewable and biodegradable, H_2_ production by fermentation of this material is potentially competitive over conventional process [5] and technically more feasible than other biological methods, including photofermentation and photobiolysis. Furthermore production of H_2_ from organic substrates is viewed as an environmentally friendly process because of its potential to yield clean energy while reducing waste and greenhouse gas emissions. Although a more detailed life cycle assessment of feedstock materials is required to fully understand the environmental impact of the whole process, the possible implications on climate change have prompted growing attention to the fermentative production of H_2_ in recent years [10].

Chemotrophic H_2_ production can be operated at mesophilic (25–40 °C), thermophilic (40–65 °C) or hyperthermophilic (>80 °C) temperatures [11,12,13], but the process in heated cultures benefits from thermodynamically favorable reactions [14,15]. Although metabolic activity sharply drops outside the optimum temperature range, increase of temperature accelerates reaction rates and offers a number of technical advantages including reduction of viscosity, improvement of mixing efficiency, reduced risk of contamination and no need for reactor cooling [16]. In addition, the high operating temperature enhances hydrolysis rate of complex substrates and, generally speaking, thermophiles can more effectively utilize complex sugars, e.g., cellulose, than mesophiles [17,18]. Furthermore, hyperthermophilic conditions suffer less from inhibition due to H_2_ partial pressure and, in the case of microbial consortia, are less sensitive to H_2_ consumers like methanogens [19,20].

In the last years, pure cultures of the hyperthermophilic eubacterium *Thermotoga neapolitana* has shown promising results for fermentative H_2_ production from several organic substrates [21]. In a recent paper [22], we have also shown that *T. neapolitana* can yield significant amounts of lactic acid without affecting H_2_ synthesis, thus offering novel applications for the fermentative process. Here we critically review the most recent data on H_2_ production by *T. neapolitana* and discuss the challenges and future prospects of H_2_ production using this bacterium.

## 2. Taxonomy of *Thermotoga neapolitana*

Originally isolated from shallow submarine hot spring nears Lucrino in the Bay of Naples in 1986 [23,24], *T. neapolitana* is a gram-negative bacterium that grows between 55 and 90 °C with an optimal growth temperature of 80 °C [23,24]. The species belongs to the order *Thermotogales* (Phylum *Thermotogae*, *class* *Thermotogae*) that have, until the recent report of *Mesotoga* *prima* [25], been exclusively comprised of thermophilic or hyperthermophilic organisms. The order includes an assembly of rod-shaped, non-sporulating bacteria that are characterized by an unconventional outer envelope called the “toga”, which forms a large periplasmic space at the poles of each rod [26,27,28]. Although it has been shown that these regions could be involved in the formation of multicellular rods [29], the physiological role of the large periplasm remains unknown. *Thermotogales* also synthesizes many polysaccharide hydrolases, some exposed on the cell surface, that allow utilization of diverse sources of carbon [30,31,32,33,34,35,36,37,38,39].

The phylogenetic position of *Thermotogae* is still unresolved, even if many studies agree to place members of this phylum among the deepest branches of bacteria, and, thus, as prime candidates for evolutionary studies [21,40]. Based upon different phylogenetic approaches, the class *Thermotogae* is divided into three orders (*Thermotogales*, *Kosmotogales* and *Petrotogales*) containing four families (*Thermotogaceae*, *Fervidobacteriaceae*, *Kosmotogaceae* and *Petrotogaceae*) and 10 genera (*Thermotoga*, *Thermosipho*, *Fervidobacterium*, *Geotoga*, *Petrotoga*, *Marinitoga*, *Thermococcoides*, *Kosmotoga*, *Oceanotoga*, and *Defluviitoga*). The genus *Thermotoga* currently includes eleven species, *i.e.*, *T. maritima*, *T. neapolitana*, *T. thermarum*, *T. elfii*, *T. subterranea*, *T. hypogea*, *T. petrophila*, *T. naphthophila*, *T. lettingae*, *T. caldifontis*, and *T. profunda*, that thrive in marine hydrothermal vents, oil reservoir sites and volcanic springs [21]. Recently, Bandhari and Gupta [41] proposed to split the current genus *Thermotoga* into two evolutionary distinct groups. According to this last classification, the original genus *Thermotoga* retains only the species *T. maritima*, *T*. *neapolitana*, *T. petrophila*, *T. naphthophila*, *Thermotoga* sp. EMP, *Thermotoga* sp. A7A and *Thermotoga* sp. RQ2 while the other *Thermotoga* species (*T. lettingae*, *T. thermarum*, *T. elfii*, *T. subterranea* and *T. hypogea*) belong to the new genus *Pseudothermotoga* [41].

## 3. Dark Fermentation Pathway in *Thermotoga neapolitana*

Chemotropic production of H_2_ is a respiration process using H^+^ as electron acceptor. The biochemical synthesis by bacteria of the genus *Thermotoga* entails catabolism of carbohydrates even if different members of the genus have the ability to use a large variety of substrates that, for example in *T. elfii*, include also sulfur compounds [42]. As in the related species *T. maritima* [43,44], *T. neapolitana* harvests energy mainly by glycolysis via the Embden-Meyerhoff pathway (EMP) [45]. EMP is the most common route for oxidation of glucose (and other hexoses) and supplies energy (ATP), reducing equivalents (NADH) and pyruvate, that undergoes terminal oxidation (acetate) or is used for biosynthesis (e.g., acetyl-CoA). According to the classical model of fermentation, generally referred to as Dark Fermentation (DF), 4 mol of H_2_ can be theoretically produced per mole of consumed glucose [46]. This molar ratio between H_2_ and glucose is usually referred to as the Thauer limit and represents the highest yield that can be achieved by a sugar-based fermentation by thermophilic bacteria. As fermentative H_2_ production is a mean to dispose of electrons, there is a direct relationship between the biogas yield and the type of the organic products that are concurrently released during the process. Yield is optimized only when all glucose is converted to acetate because NADH and electrons are fully consumed to produce the energy carrier (Equation (1)). On the other hand, in a redox neutral process, no H_2_ is produced when lactic acid is the organic product released in the medium (Equation (2)).

(1) $${\text{C}}_{6}{\text{H}}_{12}{\text{O}}_{6}+4\text{ADP}+4\text{Pi}\to 2{\text{CH}}_{3}{\text{CO}}_{2}\text{H}+2{\text{CO}}_{2}+4{\text{H}}_{2}+4\text{ATP}+2{\text{H}}_{2}\text{O}$$

(2) $${\text{C}}_{\text{6}}{\text{H}}_{\text{12}}{\text{O}}_{\text{6}}\text{ + 2ADP + 2Pi}\to 2{\text{CH}}_{\text{3}}{\text{CH(OH)CO}}_{\text{2}}{\text{H + 2ATP + 2H}}_{\text{2}}\text{O}$$

**Figure 1:**
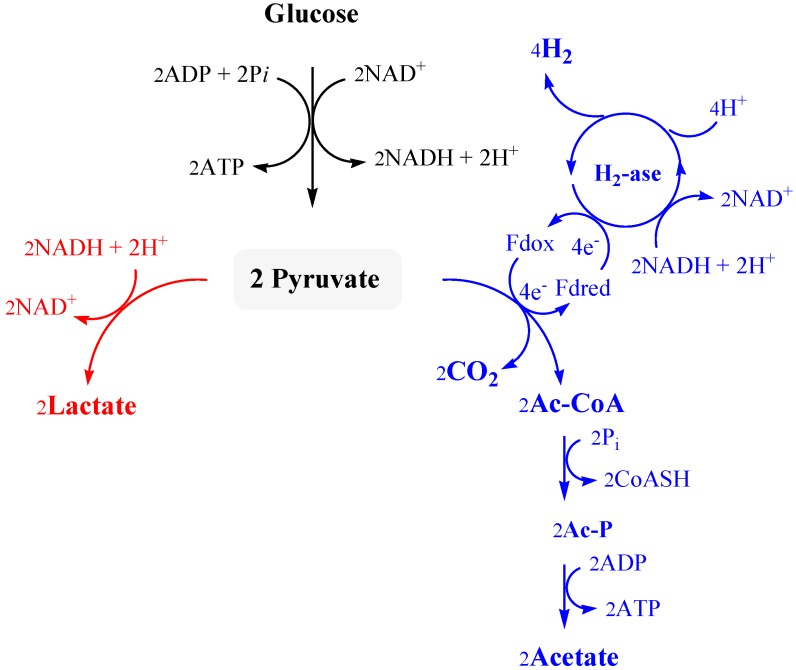
Streamlined biochemical pathway for fermentative H_2_ production, adapted from Reference [22]. Water is omitted for simplicity.

As shown in Figure 1, acetate production is driven by formation of additional ATP but, when H_2_ accumulates and consumption of NADH stops, pyruvate is diverted away for the synthesis of other organic substrates, mostly lactate that is produced by lactate dehydrogenase (LDH) with the concomitant oxidation of NADH. Lactate levels reported during fermentation by *Thermotoga* species vary from trace amounts up to levels rivaling those of acetate [44,47,48,49]. Low levels of alanine and ethanol have been also reported in *T. neapolitana* [21,45,50].

The mechanism behind the high H_2_ yields achieved by *T.* *neapolitana* is likely related to the unique characteristic of the heterotrimeric [FeFe]-hydrogenase that is present in the bacterium. Hydrogenases (H_2_ase) constitute a family of enzymes that efficiently reduce protons to H_2_ in many anaerobic microorganisms. Sequence analysis on the three proteins that form the H_2_ase of *T. maritima*, which has more than 90% homology with that of *T. neapolitana*, suggests that the β subunit is a flavoprotein that accepts electrons from NADH, and the γ subunit transfers electrons from the β subunit to the catalytic α subunit. The catalytic site (the so-called H cluster) shows the most complex Fe-S structure characterized to date and requires the specific action of three highly conserved proteins to be assembled [51]. Despite the detailed knowledge of the active site, how the endergonic reaction of H_2_ production is accomplished under physiological conditions is not clear. In fact, the reduction of H_2_ase by NADH is an energetically unfavorable reaction and the reaction is typically influenced by environmental conditions such as pH, cell growth rate and H_2_ partial pressure. In many thermophilic bacteria and several *Clostridium* species, the transfer of electrons to proton ion by [FeFe]-H_2_ase requires the presence of NADH-Ferredoxin oxidoreductase (NFOR). In this reaction, it is suggested that the oxidized ferredoxin (Fd) is reduced by NADH, which is formed during carbon metabolism. Then, the electrons in Fd are transferred to protons by [FeFe]-H_2_ase to form molecular H_2_ (Figure 1) [52].

Recently Schut and Adams proposed a novel model for H_2_ production for *Thermotoga* species based on the synergistic effect of NADH and reduced Fd [44]. According to this study, H_2_ase of *T. maritima* concurrently oxidizes reduced Fd and NADH in a 1:1 ratio in order to reduce the H^+^ ions. Ferredoxin is cyclically produced by pyruvate Fd oxidoreductase (POR) during oxidation of pyruvate to acetyl coenzyme A (Figure 1). This mechanism that couples an exoergonic reduction with an endoergonic reduction has been called “bifurcating” [53] and it is proposed to correspond to a novel type of energy conservation. Thus, energy from the oxidation of Fd drives the unfavorable oxidation of NADH in *T. maritima* [44] and the hyperthermophilic bacterium has the ability to achieve H_2_ yields close to the Thauer limit. According to this mechanism, H_2_ production by H_2_ase of *T. neapolitana* is influenced by factors that affect either NADH or reduced Fd. Furthermore, the composite mechanism of this H_2_ase is consistent with the complexity of the trimeric structure, which is much greater than that of the typical Fd-dependent, single subunit [Fe-Fe]-H_2_ase found in *Clostridium* spp. [44].

## 4. Production of Lactic Acid and H_2_ by Capnophilic Lactic Fermentation

*T. neapolitana* and the other taxonomically-related species, such as *T. maritima*, *T. petrophila*, *T. naphtophila*, *T. caldifontis*, *T. profunda*, *Pseudothermotoga thermarum*, *P. elfii*, *P. subterranea*, *P. hypogea* and *P. lettingae* have been targeted for biological production of H_2_ because of yields approaching the theoretical maximum value (Thauer limit) of 4 mol H_2_/mol glucose [46]. According to Figure 1, this result can be achieved only if all of the reducing equivalents from glucose oxidation are used to reduce protons to H_2_. Nevertheless, as discussed above, in practice these reducing equivalents are also employed for biosynthetic purposes or formation of other fermentation products. Thus, the high H_2_ yields and low production of biomass that have been reported for *T. neapolitana* suggest that pyruvate is only partially used in other metabolic transformations under standard operating conditions [54].

Inflow of gases is the most commonly reported method for removing oxygen and H_2_ from bacterial cultures in closed reactors [55,56]. Use of CO_2_ as gas sparging significantly increases the rate of both glucose consumption and hydrogen production even if there was no improvement of the overall productivity and molar yield that remained substantially unchanged in comparison with N_2_ [22]. Paradoxically, CO_2_ stimulated also synthesis of lactic acid. Feeding experiments with labeled precursors clearly proved that at least part of exogenous CO_2_ is biologically coupled with acetyl-CoA to give lactic acid when the cultures are stripped by CO_2_ gas or enriched in sodium bicarbonate. The process recycles glycolysis-derived acetyl-CoA or employs exogenous acetate with ATP consumption. In this latter case the overall outcome is a conversion of equimolar concentration of acetate and carbon dioxide into lactic acid according to reaction Equation (3).

(3) $${\text{CH}}_{\text{3}}{\text{CO}}_{\text{2}}{\text{H + CO}}_{\text{2}}{\text{ + 4H}}^{+}+4e^{-}\to {\text{CH}}_{\text{3}}{\text{CH(OH)CO}}_{\text{2}}{\text{H + H}}_{\text{2}}\text{O}$$

**Figure 2:**
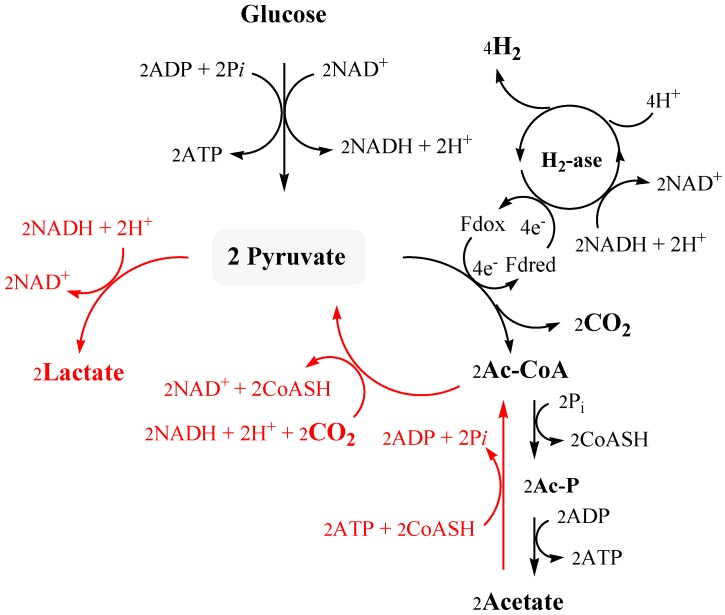
Proposed model of capnophilic lactic fermentation, adapted from [57]. Water is omitted for simplicity.

The fermentative CO_2_-dependent synthesis of lactic acid and hydrogen was named capnophilic lactic fermentation (CLF) and, as suggested in Figure 2, it put forward the possibility to fully convert sugar to lactic acid (or other reduced derivatives of pyruvate) without affecting hydrogen synthesis by means of an additional consumption of reducing equivalents deriving from other cellular processes [57].

To date, CLF has been described only in *T. neapolitana* but the pathway is likely to occur in other species of the order *Thermotogales*. The key enzyme of the process is a Pyruvate Synthase (also named Pyruvate Oxido-Reductase) that utilizes reduced ferredoxin as source of electrons [57]. In *Thermotogales* reductive carboxylation of Ac-CoA likely requires the pool of Fd that is also involved in hydrogen production. Role of Fd as efficient reductant in pyruvate synthesis has been demonstrated *in vitro* with *Clostridium thermoaceticum* [58] and suggested *in vivo* for methanogenic archaea, such as *Methanosarcina barkeri* [59]. It is noteworthy that the sequence of pyruvate oxido-reductase of this last organism has a good relation to those of *T. neapolitana* and *T.* *maritima* [57]. CLF is an example of biological sequestration of carbon dioxide by coupling with an exogenous substrate (acetate, glucose, *etc.*) and release of the end-product (lactic acid) outside of the cell. Since *T. neapolitana* does not convert CO_2_ to the reduced organic compounds required for cell metabolism, the above mechanism is not related to the autotrophic fixation known in other anaerobes. In fact, unlike known autotrophic [60,61] and heterotrophic [62,63] pathways for carbon dioxide assimilation, the capnophilic metabolism of *T. neapolitana* implies complete excretion of CO_2_ after fixation in lactic acid and no synthesis of reduced organic compounds required for cell metabolism.

## 5. Substrate Metabolism by *Thermotoga neapolitana*

As discussed above, extreme thermophiles are capable of producing H_2_ yields close to the theoretical Thauer limit of 4.0 mol H_2_/mol of glucose. In addition, the theoretical maximum yield for xylose, sucrose and glycerol are 3.33 mol H_2_/mol xylose, 8.0 mol H_2_/mol sucrose and 3.0 mol H_2_/mol glycerol under dark fermentation. Glucose is the substrate that gives the highest production of H_2_ with *T. neapolitana*. In batch experiments with this sugar, independent studies have reported H_2_ yield higher than 3.5 mol/mol and production rate ranging from 23 to 50 mL/L/h at pH of 7.5 and temperature of 80 °C [45,64]. As already mentioned above, the bacterium can also efficiently use a wide range of other substrates ranging from simple to complex sugars including ribose, xylose, fructose, sucrose, maltose, lactose, galactose, starch, and glycogen (Table 1) [21,24,50,65,66,67,68,69,70,71].

Waste glycerol from bio-diesel manufacturing is currently considered an attractive and abundant feedstock for fermentation process. Batch tests conducted by Maru *et al.* [69] have demonstrated that 2.65 mol of H_2_ can be produced per mole of glycerol by using the bacterium *T. neapolitana* at a glycerol concentration of 2.5 g/L. Ngo and Sim [72] also reported that the bacterium transforms pure glycerol and crude waste glycerol with approximately similar H_2_ production (447 ± 22 and 437 ± 21 mL/L, respectively). According to these authors, these yields are better than those reported with mesophilic bacteria [73,74] and addition of itaconic acid to buffer the culture medium further increased this productivity with both substrates. It is notable that a prediction model built on comparative analysis of the genomes of *T. maritima* and *T. neapolitana* put forward that this latter species should not be able to metabolize a number of sugars, including cellotetraose. However, experimental assessment proved that the bacterium grows on this substrate despite that the model predicted an incomplete cellotetraose transport complex. Proteomic analysis of glucose and cellotetraose revealed two possible new gene clusters that may be associated with transport of these sugars [75].

Cappelletti *et al.* [65] showed that *T. neapolitana*, *T. maritima*, *T. petrophila* and *T. naphtophila* produce about 2.95 mol of H_2_ per mol of glucose equivalent from molasses and 2.5 mol of H_2_ per mol of glucose equivalent from cheese whey, whereas 2.7–2.8 mol of H_2_ per mol of glucose equivalent were produced on carrot pulp hydrolysates containing glucose, fructose and sucrose as main sugars [50]. Enzymatic hydrolysis of the polysaccharide fraction prior to fermentation increased the H_2_ yield of almost 10% to 2.3 g/kg of hydrolyzed carrot pulp. Lignocellulosic substrates (e.g., crop residues) were tested for H_2_ production with some standard pretreatment to wash out lignin [70,76,77]. Thermo-chemical pretreatment (*i.e.*, heat, ammonia soaking and dilute H_2_SO_4_ soaking) were found to be effective pretreatment techniques to remove lignin and enhance availability of simple sugars for H_2_ production. According to Ngo *et al.* [76] 2.8 mol of H_2_ per mol of xylose equivalent are produced by *T. neapolitana* in a pH-controlled continuously stirred anaerobic bioreactor sparged with N_2_ gas. Similar results have also been reported with rice straw pretreated with ammonia soaking and diluted sulfuric acid [70]. Algal biomass (*Chlamydomonas reinhardtii*) pretreated by heat-HCl or Termamyl^®^ enzymatic hydrolysis has been also used as substrate of *T. neapolitana* to give 2.5 mol of H_2_ per mol of glucose equivalent [71]. Without pretreatment, a slightly lower yield (2.2 mol/mol of glucose equivalent) was produced by fermentation of laminarans derived from the marine diatom *Thalassiosira weissflogii* [66].

**Table 1:** H_2_ production from various substrates by hyperthermophilic eubacterium *T. neapolitana*. B = batch; FB = fed-batch; AA = Acetic acid; LA = Lactic acid; EtOH = Ethanol.

Carbon Source	Substrate Load (g/L)	Culture Type	T(°C)/Start pH	Mixing Speed (rpm)	Reactor Volume (mL)	Working Volume (mL)	H_2_ Yield	Byproducts	Ref.
Glucose	5	B	80/7.5	250	3800	1000	2.8 mol H_2_/mol glucose	AA, LA, CO_2_	[22]
Glucose	5	B	80/7.1	250	2400	600	3.5 ± 0.1 mol H_2_/mol glucose *^a^*	AA, LA, CO_2_	[45]
Glucose	10	B	72/7.0	350	2000	1000	3.5 mol H_2_/mol glucose	AA, LA, CO_2_	[50]
Glucose	20	B	72/7.0	350	2000	1000	3.4 mol H_2_/mol glucose	AA, LA, CO_2_	[50]
Glucose/Fructose 7:3	10	B	72/7.0	350	2000	1000	3.3 mol H_2_/mol glucose	AA, LA, CO_2_	[50]
Glucose/Fructose 7:3	20	B	72/7.0	350	2000	1000	3.0 mol H_2_/mol glucose	AA, LA, CO_2_	[50]
Fructose	10	B	72/7.0	350	2000	1000	3.4 mol H_2_/mol fructose	AA, LA, CO_2_	[50]
Fructose	20	B	72/7.0	350	2000	1000	3.2 mol H_2_/mol fructose	AA, LA, CO_2_	[50]
Carrot pulp hydrolysate	10	B	72/7	350	2000	1000	2.7 mol H_2_/mol glucose	AA, LA, CO_2_, EtOH	[50]
Carrot pulp hydrolysate	20	B	72/7	350	2000	1000	2.4 mol H_2_/mol glucose	AA, LA, CO_2_, EtOH	[50]
Glycerol	5	B	75/7.5	-	120	40	2.7 ± 0.1 mol H_2_/mol glycerol	AA, LA, CO_2_	[64]
Molasses	20	B	77/8.5	100	116	40	2.6 ± 0.1 mol H_2_/mol glucose	AA, LA, CO_2_	[65]
Cheese whey	12.5	B	77/8.5	100	116	40	2.4 ± 0.1 mol H_2_/mol glucose	AA, LA, CO_2_	[65]
Diatom *^b^* water soluble sugars	2	B	80/7.5–8	250	3800	500	1.9 ± 0.1 mol H_2_/mol glucose	AA, LA, CO_2_	[66]
Glucose	5	B	80/8.0	200	120	60	3.8 ± 0.4 mol H_2_/mol glucose	AA, LA, CO_2_	[67]
Arabinose	5	B	80/8.0	200	120	60	3.8 ± 0.5 mol H_2_/mol arabinose	AA, LA, CO_2_	[67]
Xylose	5	B	80/8.0	200	120	60	3.4 ± 0.3 mol H_2_/mol xylose	AA, LA, CO_2_	[67]
Potato steam peels	10	B	75/6.9	350	2000	1000	3.8 mol H_2_/mol glucose	AA, LA, CO_2_	[68]
Glycerol	2.5	B	80/7.3	200	120/240	25/50	2.6 mol H_2_/mol glycerol	AA, LA, CO_2_	[69]
Rice straw	10	B	75/7.5	150	120	40	2.7 mmol H_2_/g straw	-	[70]
Algal *^c^* starch	5	B	75/7–7.4	150	120	40	2.5 ± 0.3 mol H_2_/mol glucose	-	[71]
Glycerol	1–10	B	75/7.5	-	120	40	620 ± 30 mL H_2_/L glycerol	AA, LA, CO_2_	[72]
Xylose	5	B	75/7.5	300	3000	1000	2.8 ± 0.1 mol H_2_/mol xylose	AA, LA, CO_2_	[76]
Glucose/Xylose 7:3	10–28	B	80/6.8	350	2000	1000	2.5–3.3 mol H_2_/mol glucose	AA, LA, CO_2_	[77]
Cellulose	10–28	B	80/6.8	350	2000	1000	2.0–3.2 mol H_2_/mol glucose	AA, LA, CO_2_	[77]
Xylose	5	B	75/7.0	300	3000	1000	1.8 ± 0.1 mol H_2_/mol xylose	AA, LA, CO_2_	[78]
Glucose	7.5	B	77/8.5	100	119	40	1.3 ± 0.1 mmol H_2_/g glucose	AA, LA, CO_2_	[79]
Molasses	20	B	77/8.5	100	119	40	1.8 ± 0.1 mol H_2_/g glucose	AA, LA, CO_2_	[79]
Cheese whey	12.5	B	77/8.5	100	119	40	1.04 ± 0.05 mol H_2_/mol glucose	AA, LA, CO_2_	[79]
Glucose	5	FB	75/7.5	300	3000	1000	3.2 ± 0.2 mol H_2_/mol glucose	AA, LA, CO_2_	[80]
Xylose	5	FB	75/7.5	300	3000	1000	2.2 ± 0.1 mol H_2_/mol xylose	AA, LA, CO_2_	[80]
Sucrose	5	FB	75/7.5	300	3000	1000	4.9 ± 0.2 mol H_2_/mol sucrose	AA, LA, CO_2_	[80]
Glucose	2.5	B	77/7.5	75	160	50	3.8 ± 0.3 mol H_2_/mol glucose	AA, LA, CO_2_	[81]
Glucose	5	B	70/8.5	75	160	50	24% H_2_ (*v*/*v*) headspace *^d^*	CO_2_	[82]
Glucose	7	B	77/7.5	150	120	40	3.2 ± 0.1 mol H_2_/mol glucose	AA, LA, CO_2_	[83]
Xylose	4	B	77/7.5	150	120	40	2.2 ± 0.1 mol H_2_/mol xylose	AA, LA, CO_2_	[83]
Glucose	5	B	70/8.5	-	160	50	25%–30% H_2_ (*v*/*v*) headspace *^d^*	AA, LA, CO_2_	[84]
Cellulose *^e^*	5	B	75–80/7.5	150	120	50	0.25 ± 0.01 mol H_2_/mol glucose	AA, CO_2_	[85]
Cellulose derivative	5	B	75–80/7.5	150	120	50	0.77 ± 0.04 mol H_2_/g glucose	AA, CO_2_	[85]
Cellulose	5	B	80/7.5	150	120	40	2.2 mol H_2_/mol glucose	AA, CO_2_	[85]
Starch	5	B	75–80/7.5	150	120	50	1.4 ± 0.1 mL H_2_/g glucose	AA, CO_2_	[85]

*^a^* excluding the estimated contribution from protein; *^b^* *Thalassiosira weissflogii*; *^c^* *Chlamydomonas reinhardtii*; *^d^* yield not reported; *^e^* *Miscanthus giganteus*.

There are conflicting reports on the effect of protein lysates on growth and H_2_ production by *T. neapolitana*. Maru *et al.* [69] noticed that reduced level of yeast extract (YE) negatively affects H_2_ production but increasing concentration from 2 to 4 g/L did not induce significant change in gas evolution. On the contrary, increasing YE concentration from 1 to 4 g/L improved biomass and H_2_ production in cultures of *T. neapolitana* on glycerol as reported by other independent studies [72,73]. No effect is reported through increasing the concentration of protein lysates above 5 g/L. Cappelletti *et al.* [65] reported that partial production of H_2_ can be due to metabolism of tryptone soy broth (TSB) whereas the contribution of YE is null. On the other hand, transformation of peptone, tryptone and YE yields 10%–15% to the total H_2_ production according to d’Ippolito *et al.* [45] and Eriksen *et al.* [67].

## 6. Systems Integration

According to Levin *et al.* [86], H_2_ production by dark fermentation is considered the most practically applicable process for production of the energy carrier. However, as shown in Figure 1, only 2 mol of carbon from the substrate (*i.e.*, glucose) are fully oxidized to CO_2_ and only 4 mol H_2_ are formed. Thus, according to the dark fermentation model, a fermentative H_2_ production can only convert, even in an optimal condition, less than 33% of the energy from the substrate (e.g., glucose). On the other hand, the transformation efficiency can be significantly improved (theoretically up to 12 mol H_2_ per mol of glucose) if a second biological process allows for the complete oxidation of the residual products released by the thermophilic process. In particular, photo-heterotrophic fermentation of organic acids produced by *T. neapolitana* is hypothetically entitled to produce a further 8 mol of H_2_, thus reaching the maximum possible yield of 12 mol of H_2_.

Purple nonsulphur (PNS) bacteria are a non-taxonomic group of microorganisms that are attractive for the biological production of H_2_ from biomass (reviewed in [87]). A few studies have also demonstrated that these microorganisms can be successfully integrated into a two-step process to produce H_2_ in combination with dark fermentation. The first report of a two-stage process with *T. neapolitana* by Uyar and coworkers [88] showed that *Rhodobacter capsulatus* effectively produces hydrogen when the concentration of acetate is lower than 60 mM in the spent medium of the thermophilic bacterium. Interestingly these authors also noticed that addition of iron II in the range of 0–29 µM to the culture medium (*i.e.*, to the spent medium of thermophilic bacterium) of *R. capsulatus* increased the hydrogen production in a significant manner (1.37 L H_2_/L culture in effluent media supplemented with iron and vitamins; 0.30 L H_2_/L culture in effluent media supplemented only with vitamins). More recently, we have repeated the experiment with *T. neapolitana* and *Rhodopseudomonas palustris* by replacing the traditional conditions of DF (dark fermentation) with those of CLF (capnophilic lactic fermentation) [89]. To achieve photo-fermentation by a mutant strain of *R. palustris* [90], *T. neapolitana* was grown under reduced level of NaCl and nitrogen-containing compounds. According to Uyar *et al.* [88], the spent broths of the thermophilic bacterium were only supplemented with Fe-citrate and phosphate buffer. The combined microbial system gave 9.4 mol of hydrogen per mole of glucose consumed during the anaerobic process, which is the best production yield so far reported for conventional two-stage batch cultivations [89]. The results also proved that CLF can be used for inducing a metabolic switch in *T. neapolitana* that brings actual improvements of hydrogen yields in combination with photofermentation.

The advantages of using biomass for H_2_ production range from the mitigation of CO_2_ and other pollutant emissions, to reduction of environmental and economical costs for disposing wastes. Limitations in use of biomass are mainly due to the seasonal availability of agro wastes, costs of their collection and incomplete use of the organic matter. In this view, microalgal biomass is an attractive alternative since algal cultivation can theoretically run continuously with no restriction due to seasonal cycle and can yield large amounts of biomass of constant composition. Furthermore, fermentation of algal feedstock can be associated with production of biofuels or by-products of high value. *T. neapolitana* directly produces H_2_ by fermentation of the biomass of the green alga *Chlamydomonas reinhardtii* with molar yields (1.8–2.2 mol/mol glucose equivalent) depending on pretreatment methods [71]. *T. neapolitana* possesses genes encoding both for a 1,3-β-glucosidase BglB (laminaribiase) and a 1,3-β-glucanase LamA (laminarinase) that are able to completely degrade chrysolaminarin, the storage polysaccharides of diatoms, to glucose with a synergic action [91,92]. Accordingly, the bacterium fermented the water-soluble fraction of the marine diatom *Thalassiosira weissflogii* without any pretreatment [66]. Production (434 mL/L in 24 h; 18.1 mL/L/h) and yield (2.2 mol H_2_/mol glu. eq) of H_2_ on diatom extracts containing 2 g/L of sugar equivalent were just slightly lower than those achieved by fermentation of glucose (809 mL/L in 24 h; 33.7 mL/L/h; 3.0 mol H_2_/mol glu) and pure chrysolaminarin (643 mL/L in 24 h; 26.8 mL/L/h; 3.2 mol H_2_/mol glu. eq).

## 7. Bioreactor Configuration

Several bioreactor configurations such as continuously stirred tank reactors (CSTRs), fluidized bed reactors (FBRs), packed bed reactors (PBRs), up-flow anaerobic sludge blanket (UASB) reactors, anaerobic sequencing batch reactors (AnSBRs), high rate/hybrid reactors, and membrane biological reactors (MBRs) have promising prospects for dark fermentation processes [14]. Table 2 reports the CSTR used for fed-batch and continuous reactors studied for H_2_ production by suspended and immobilized cells of *T. neapolitana*. CSTRs operate continuously and the bulk inside the reactor is mixed uniformly. However the mixing rate depends on the reactor geometry and power input [93]. CSTRs favor mass transfer among biomass, substrates and gases, and are effective in temperature and pH bulk control. However, CSTR can experience biomass washout, when the loading and the dilution rate increases.

The biomass washout is less probable in attached biomass reactors (e.g., FBRs, PBRs, UASB) where inert material is used to support and contain the bacteria, thus providing a high concentration of cells and, consequently high solids retention time, high organic load, high mass transfer efficiency and high tolerance for shock loads [78,94,95]. Several inert materials have been used successfully as support for *T. neapolitana* growth, *i.e.*, coir, bagasse, loofah sponge, expanded clay, diatomaceous clay, activated carbon, polysaccharide gels (e.g., alginate, k-carrageenan, agar, chitosan), synthesized materials (e.g., polyvinyl alcohol (PVA), silicone, polyacrylamide, urethane foam and polymethyl methacrylate) [26,96,97,98,99], ceramic porous carries (*i.e.*, biomax) [65,79], and porous glass beads [78]. The batch fermentation tests conducted by Ngo and Bui have shown that the H_2_ production rate and H_2_ yield of the immobilized cells reached the highest values of 5.64 ± 0.19 mmol H_2_/L/h and 1.84 ± 0.1 mol H_2_/mol xylose, respectively, which were 1.7- and 1.3-fold higher than those with free cells [78]. Synthetic hydrogels based on methacrylate derivatives with buffer capacity also effectively supported cell growth and hydrogen production [96,100]. In particular, the use of hydrogel with positive charge and amine groups doubled hydrogen production rate compared with suspension cultures. Both sugar metabolism and hydrogen synthesis were affected positively by neutralization of the acidic side-products of the fermentation, *i.e.*, acetate and lactate. The presence of positively charged groups on the inert support proved to be critical to promote the colonization of the polymeric material by a great number of *T. neapolitana* cells laying in a biofilm-like arrangement.

**Table 2:** Continuous and fed-batch operation in CSTRs for *T. neapolitana*.

Substrate	Reactor Volume (L)	Working Volume (L)	Temp. (°C)	Culture Type	Culture Condition	H_2_ Yield	References
Glucose/Xylose/Arabinose	3.0	2.75	80	Suspended cells	Fed-batch	3.8 ± 0.4 mol H_2_/mol glucose; 3.4 ± 0.3 mol H_2_/mol xylose; 3.8 ± 0.5 mol H_2_/mol arabinose	[67]
Glucose/Sucrose/Xylose	3.0	1.0	75	Suspended cells	Fed-batch	3.2 ± 0.16 mol H_2_/mol glucose; 4.95 ± 0.25 mol H_2_/mol sucrose; 2.2 ± 0.11 mol H_2_/mol xylose	[80]
Xylose	3.0	1.0	75	Immobilized cells	Fed-batch	1.84 ± 0.1 mol H_2_/mol xylose	[78]
Glucose/Cheese whey/Molasses	19.0	15.0	77	Suspended cells	Continuous	1.2 mmol H_2_/L/h for glucose; 0.42 mmol/L/h for cheese whey; 1.3 mmol/L/h for molasses	[79]
Glucose	-	-	80	Immobilized cells	Fed-batch	3.3 mol H_2_/mol glucose	[96]

## 8. Operating Conditions and Kinetics of *Thermotoga neapolitana* Fermentation

### 8.1. Hydraulic Retention Time (HRT)

In the dark fermentative H_2_ production, hydraulic retention time (HRT), organic loading rate (OLR) and pH are coupled variables since short HRT and high OLR generally correspond to low pH condition that affects the biomass metabolism. Both high OLR and low HRT represent favorable conditions for H_2_ production as such operating conditions inhibit other slow growing bacteria, such as methanogens [101]. A HRT in the range of 0.25–60 h (lower HRT for attached/immobilized biomass and higher HRT for suspended growth biomass) is proved to be suitable for hyperthermophilic dark fermentative H_2_ production by *T. neapolitana* in batch, fed-batch and continuous bioreactors using a wide range of substrates such as glucose, sucrose, starch, lignocellulose, organic waste and algal starch [102]. *T. neapolitana* is an exceptionally robust microorganism for H_2_ production because of its efficient hydrolytic abilities and adaptability to different culture conditions [77]. Nevertheless, production of H_2_ is optimal only in very restricted range of operating conditions. In particular, the bacterium grows in a wide interval of temperatures (*i.e.*, 55–90 °C) but the highest H_2_ production occurs between 75 and 80 °C [76].

### 8.2. Working pH

As reported above, culture pH is directly affected by the acidogenic activity. Consequently, pH control by base addition (e.g., NaOH) is critically important to maximize both H_2_ production and substrate consumption [45,80,81]. Growth of *T. neapolitana* is inhibited at pH of 4.5 [82], whereas change from 4.0 to 5.5 induces an increase of H_2_ content in the headspace from 42% to 64% [103]. Nguyen *et al.* demonstrated that variation of pH in *T. neapolitana* cultures from 5.5 to 7.0 enhances cumulative H_2_ production from 125 to 198 mL H_2_/L medium, but further increase to 8.0–9.0 leads to total decline in the biogas evolution [83]. At laboratory scale, the strict control of pH has also suggested the use of compounds with increased buffer capacity such as diacid/monoacid phosphate (HPO_4_^−2^/H_2_PO_4_^−^), tris (hydroxymethyl) aminomethane (TRIS), 3-(*N*-morpholino) propanesulfonic acid (MOPS), piperazine-*N*,*N*′-bis(2-ethanesulfonic) acid (PIPES), and 4-(2-hydroxyethyl)-1-piperazineethanesulfonic acid (HEPES) [77,81,82,84]. For large scale application the use of these chemicals is probably economically prohibitive but similar effects could be achieved by use of more convenient products (e.g., CO_2_).

### 8.3. Temperature

The primary fermentation products for *T. neapolitana* across the permissive growth temperature range are H_2_, CO_2_, acetate and small amounts of lactate. Two independent studies on the influence of temperature on H_2_ production of *T. neapolitana* and *T. maritima* support a direct correlation with H_2_ production and bacterial growth [81,85]. Munro *et al.* reported that rate and amount of glucose consumption and H_2_ formation increased by arising the operating temperature from 60 to 77 °C, but there was no significant difference from 77 to 85 °C [81]. Although production of acetate and lactate indicated a difference between 77 and 85 °C, a comparison of the molar yields acetate/glucose and lactate/glucose for the operating temperatures between 65 and 85 °C suggested no significant change in molar yield for the two organic acids.

### 8.4. Partial Pressure

The total and partial pressure of gas inside the reactor influences the biomass growth and product formation. According to Schonheit and Schafer [104], H_2_ itself inhibits the process in a batch reactor. Van Niel *et al.* reported that H_2_ partial pressure less than 20 kPa is required for reactor operating at high temperature (>70 °C) [20]. Partial pressure of H_2_ above 20 kPa reverses the metabolic pathway, thereby facilitating the production of more reduced products such as acetone, ethanol, lactate, butanol and alanine [11,50,105]. Experimental data show that use of pure nitrogen as gas sparging and high ratio between headspace volume/culture volume can contain the partial pressure of H_2_ below the critical limit in cultures of *T. neapolitana* [45,67,81]. Increase in yield and production of H_2_ are reported by N_2_ sparging compared to no sparging condition [45,83]. The tolerance of *T. neapolitana* to oxygen is matter of debate. Van Ooteghem *et al.* described significant improvement of H_2_ production under microaerobic condition [82,84], whereas Eriksen *et al.*, in line with other studies, reported that *T. neapolitana* can tolerate only low oxygen partial pressure (1% or 1.2 kPa) and found that 6% O_2_ (7.2 kPa) inside the reactor completely inhibits H_2_ production and reduces glucose consumption from 12 to 4 µmol/h [106].

### 8.5. Mathematical Modeling and Kinetics

Metabolic transformation of glucose by *T. neapolitana* can be effectively described with the Equation (4).

(4) $${\text{C}}_{\text{6}}{\text{H}}_{\text{12}}{\text{O}}_{\text{6}}+\left(2-m\right){\text{H}}_{\text{2}}\text{O}\to \left(4-m\right){\text{H}}_{\text{2}}+\left(2-m\right)\text{AA}+\left(2-m\right){\text{CO}}_{2}+m\text{LA}$$

where *m* is a stoichiometric coefficient [22].

A majority of studies have either adapted or modified exiting mathematical (or empirical) models to describe the experimental results [107]. Gompertz empirical model (Equaton (5)) and International Water Association (IWA) anaerobic digestion model No. 1 (ADM1) are the most popular models to simulate dark fermentation. The Gompertz model is particularly used to estimate the maximum hydrogen production potential and to determine the lag phase for H_2_ production [108] but it does not allow the process kinetics to be addressed because of the exclusion of operating conditions (e.g., substrate type and concentration, pH, temperature, and partial pressure of gas mixture) that regulate the fermentation reaction [108].

(5) $$H(t)=P*\exp\left\{-\exp\left[\frac{R_{m}e}{P}\left(\text{λ}-t\right)+1\right]\right\}$$

where

*H* (*t*) = Cumulative H_2_ production (mL/L)
*P* = H_2_ production potential (mL H_2_)
*R_m_*= Maximum H_2_ production rate (mL H_2_/h)
*t* = Incubation/cultivation time (h)
λ = Duration of the lag phase (h)

On the other hand, ADM1 is a complete and comprehensive kinetic model based on Monod kinetic Equations (6) [109,110,111,112] and has been used often to model DF reactions [79,113,114].

(6) $$\text{μ}=\frac{{\text{μ}}_{\max}S}{k_{S}+S}$$

where

μ = specific growth rate of biomass (h^−1^)
μ_max_ = maximum specific growth rate of biomass (h^−1^)
*k_s_* = semi saturation constant (g/L); *k_s_* equals the substrate concentration at which μ equals ½ μ_max_
*S* = substrate concentration (g/L)

The ADM1-based model and Gompertz empirical model have been extensively used to study H_2_ production by fermentative process, but to date there are only two studies with *T. neapolitana*. In pure culture on glucose at 77 °C, Yu and Drapcho [114] reported maximum specific maximum growth rate (µ_max_) of 0.94 h^−1^ and semi saturation constant (*k_s_*) of 0.57 g sugar/L when H_2_ and biomass product yields were 0.0286 g H_2_/g glucose and 0.248 g biomass/g glucose, respectively. More recently, Frascari *et al.* [79] have studied the kinetic parameters for *T. neapolitana* grown on glucose, molasses and cheese whey by suspended or immobilized cells. The µ_max_ value with immobilized bacteria (0.09 ± 0.05 h^−1^ for glucose, 0.19 ± 0.02 h^−1^ for molasses and 0.042 ± 0.007 h^−1^ for cheese whey) was found to be significantly higher than with suspended cells (0.024 ± 0.005 h^−1^ for glucose, 0.055 ± 0.005 h^−1^ for molasses and 0.033 ± 0.006 h^−1^ for cheese whey). On the contrary, the semi saturation constant (*k_s_*) was 0.09 ± 0.05 g sugar/L for glucose, 0.6 ± 0.2 g sugar/L for molasses and 1.2 ± 0.3 g sugar/L for cheese whey in the immobilized systems and 1.1 ± 0.3 g sugar/L for glucose, 0.2 ± 0.05 g sugar/L for molasses and 1.5 ± 0.5 g sugar/L for cheese whey with bacterial suspensions [79].

## 9. Conclusions

Among the various technologies, fermentation has many advantages for the biological production of H_2_ and is theoretically feasible for large-scale application particularly from the fermentation of solid wastes [115,116]. Extensive research in the last decades has shown the promising prospect of using pure cultures of the bacterium *T. neapolitana*. Like other hyperthermophilic process, the technology is readily used at laboratory scale with high production rate, low energy demand, easy operation and sustainability. On the contrary little has been done in terms of comparison of cost and effectiveness between *T. neapolitana* and traditional processes that use fossil fuel for production of hydrogen.

*T. neapolitana* has also shown great potential for other applications such as recovery of byproducts with potential economic value in the market *i.e.*, lactic acid. Introduction of capnophilic process for the simultaneous production of H_2_ and lactic acid is very promising and could significantly influence the future of agro-waste management. Clearly, further research is needed to optimize the operating parameters and reactor configurations and more experiments are needed to verify process kinetics and full-scale applicability. Nevertheless, fermentation of organic material by the thermophilic bacterium could be the beachhead of a complete conversion process that generates H_2_ only as a first step.

Agro-food wastes and algal biomass seem to be attractive substrates for fermentation by *T. neapolitana* and thus are considered as feedstock for comprehensive development of biorefineries. Moreover, coupling of *T. neapolitana*-based transformation with other biological processes also seems very promising. In this view, chemotrophic production of hydrogen by hyperthermophilic bacteria has already shown great potential in association with both microalgal cultivations and photofermentation by purple nonsulphur bacteria. Finally, considerable enhancement of the fermentative capacity of *T. neapolitana* can be also expected by metabolic engineering and physiological manipulations of strains, as well as by improvement in reactor configurations [1,117]. In fact, for example the unexpected success of CLF suggests that a huge unexplored gene pool available in nature, with great potential for H_2_ production, is yet to be discovered [118].
